# *Mycobacterium tuberculosis* promotes genomic instability in macrophages

**DOI:** 10.1590/0074-02760170281

**Published:** 2018-03

**Authors:** Jorge Castro-Garza, Miriam Lorena Luévano-Martínez, Licet Villarreal-Treviño, Jaime Gosálvez, José Luis Fernández, Martha Imelda Dávila-Rodríguez, Catalina García-Vielma, Silvia González-Hernández, Elva Irene Cortés-Gutiérrez

**Affiliations:** 1 Centro de Investigación Biomédica del Noreste, Instituto Mexicano del Seguro Social, Monterrey, NL, México; 2 Universidad Autónoma de Nuevo León, Facultad de Ciencias Biológicas, Monterrey, NL, México; 3 Universidad Autónoma de Madrid, Unit of Genetics, Department of Biology, Madrid, Spain; 4 Genetics Unit, Complejo Hospitalario Universitario A Coruña, La Coruña, Spain

**Keywords:** Mycobacterium tuberculosis, alkaline-labile sites, DNA breakage detection fluorescence *in situ* hybridisation (DBD-FISH)

## Abstract

**BACKGROUND:**

*Mycobacterium tuberculosis* is an intracellular pathogen, which may either block cellular defensive mechanisms and survive inside the host cell or induce cell death. Several studies are still exploring the mechanisms involved in these processes.

**OBJECTIVES:**

To evaluate the genomic instability of *M. tuberculosis*-infected macrophages and compare it with that of uninfected macrophages.

**METHODS:**

We analysed the possible variations in the genomic instability of *Mycobacterium*-infected macrophages using the DNA breakage detection fluorescence *in situ* hybridisation (DBD-FISH) technique with a whole human genome DNA probe.

**FINDINGS:**

Quantitative image analyses showed a significant increase in DNA damage in infected macrophages as compared with uninfected cells. DNA breaks were localised in nuclear membrane blebs, as confirmed with DNA fragmentation assay. Furthermore, a significant increase in micronuclei and nuclear abnormalities were observed in infected macrophages versus uninfected cells.

**MAIN CONCLUSIONS:**

Genomic instability occurs during mycobacterial infection and these data may be seminal for future research on host cell DNA damage in *M. tuberculosis* infection.

Tuberculosis (TB) caused by *Mycobacterium tuberculosis* is one of the top 10 causes of death worldwide. In 2015, an estimated 10.4 million people were infected with TB and 1.8 million deaths reported; 35% of these deaths included HIV-positive people. Multidrug-resistant strains represent one of the major public health threats (WHO 2017).

The bacterium *M. tuberculosis* is one of the most potent pathogens, reflecting its ability to adapt to the human host. *M. tuberculosis* is preferentially an intracellular bacterium, which is able to persist within the human host for decades in a clinically latent state, wherein it is presumed to be quiescent and not replicating. Imbalances in an infected cell that cause it to either attempt to neutralise, tolerate, or repair damage caused by the cell defence system or induce cell death are incompletely understood. The cellular effectors include activated nitrogen intermediates and reactive oxygen species. These agents are able to damage a variety of cellular constituents, including DNA. Techniques for the detection and quantification of DNA damage during an infectious process may help to understand the mechanisms involved in the cell-pathogen interactions and the final outcome.

DNA breakage detection fluorescence *in situ* hybridisation (DBD-FISH) quantifies putative DNA breaks *in situ* within single cells. This technique offers the advantage of scanning the whole genome or specific DNA sequences and utilises cells that have been embedded within an inert agarose matrix on a specifically prepared microscope slide (Fernández & Gosálvez 2002, Fernández et al. 2002). The cells are lysed to remove membranes and proteins and the resultant nucleoids are exposed to a controlled denaturation step using alkaline buffers. The alkaline condition gives rise to single-strand DNA (ssDNA) stretches, which start from 5'-3' free DNA ends, or highly sensitive DNA motifs. In addition, alkaline treatment may break the sugar-phosphate backbone at basic sites or sites with deoxyribose damage, transforming these lesions into DNA breaks, which are also converted into ssDNA. These lesions are known as alkaline-labile sites (ALS).

Single-strand DNAs may be detected by hybridisation with specific or whole-genome fluorescent DNA probes. As DNA breaks increase in the target region, more ssDNAs are produced, resulting in the hybridisation of excessive DNA probes. As a consequence, an intense FISH signal is generated, which may be quantified using image analysis systems (Fernández & Gosálvez 2002, Fernández et al. 1998, 2002).

The signal from DBD-FISH obtained in the absence of exogenous DNA-damaging agents reflects the background level or constitutive DNA breaks and ALS. DNA damage levels may be reflective of the torsional stress on DNA loops associated with tight chromatin packing. It may vary between cell types in conventionally conformed genomes (e.g., sperm and lymphocytes) (Cortés-Guitiérrez et al. 2008) and may change under stress conditions, including exposure of cells to gamma irradiation (Fernández et al. 2001a) and human papillomavirus infection (Cortés-Gutiérrez et al. 2011).

The hybridisation of the whole-genome DNA probe to somatic cells may result in the background DBD-FISH signal, which is inconsistent; certain chromatin regions are selectively and strongly labelled. In addition, the DNA sequences related to constitutive ALS mostly correspond to the specific highly repetitive DNA sequences (Fernández et al. 2001a, Rivero et al. 2001). In human leukocytes, DBD-FISH areas within the genome with a more intense background correspond to DNA domains containing 5-bp satellite DNA.

Micronuclei (MN) and nuclear abnormalities are biomarkers broadly used to evaluate chromosomal instability. The detection of these biomarkers offers the opportunity to monitor cells, individuals, or populations exposed to mutagenic, genotoxic, or carcinogen events (Fenech et al. 1999).

The DNA fragmentation test is based on the removal of nuclear proteins under the principle that cells with fragmented DNA produce a characteristic halo of dispersed DNA loops, which are absent in cells with non-fragmented DNA. In addition, this method is simple and may be performed in a short period of time (Fernández et al. 2002).

Here, we aimed to use DBD-FISH to evaluate the genomic instability of macrophages infected with *M. tuberculosis* and compare it with that of uninfected macrophages.

## MATERIALS AND METHODS

*Cultures of M. tuberculosis* - The stock cultures of *M. tuberculosis* strain H37Rv used in this study were cultivated in Middlebrook 7H9 medium (Difco, Sparks, MD) with OADC (Becton Dickinson BBL, Cockeysville, MD) until mid-logarithmic phase. The bacteria were subsequently aliquoted and stored at −70°C until needed. Colony-forming units (CFUs) for each stock culture were determined by plating in triplicates on Middle-brook 7H10 agar (Carr-Scarborough, Atlanta, GA, USA). Before each experiment, aliquots of bacterial stocks were thawed at 37°C, vigorously vortexed, and serially diluted in tissue culture medium to provide inocula with a multiplicity of infection (MOI) of 5:1 (bacterium:cell). MOI was confirmed from these preparations by CFU on Middlebrook 7H10 agar for each experiment.

*Cell cultures* - Human monocyte cell line THP-1 was maintained in Roswell Park Memorial Institute (RPMI)-1640 medium (Gibco-Life technologies, Waltham, MA, USA) supplemented with 10% foetal bovine serum (FBS; Gibco-Life technologies) and 1 mM sodium pyruvate (Sigma). To transform the cells into macrophages, the cells were subcultured four times without sodium pyruvate and 1 x 10^6^ cells/well were seeded into six-well microplates (Costar Corning, New York, NY, USA) in complete RPMI-1640 medium supplemented with 10 μM phorbol-12-myristate-13-acetate (PMA; Calbiochem Biosciences, Darmstadt, Germany). Cells were washed twice with RPMI-1640 medium every 48 h for no longer than four days, followed by two washes with 1× phosphate-buffered saline (PBS). As DBD-FISH technique requires cells in suspension, macrophages were detached by their incubation with 2 mL Versene solution at 37°C for 45 min. Macrophages were harvested by centrifugation at 130x *g* for 4 min at room temperature (25-27°C), washed with 1x PBS, and finally suspended in warm (37°C) RPMI-1640 medium. Cell counting was performed using a Neubauer chamber and viability was determined by trypan blue dye.

*Infection assay* - Aliquots of 900 μL cell suspension at a final concentration of 1.1 x 10^6^ cells/mL were distributed into 2 mL screw cap tubes (Axygen, Union City, CA, USA) and 100 μL of the bacterial inoculum or RMPI-1640 medium (uninfected controls) were added to each tube. After 12 h of incubation at 37°C with 5% CO_2_, cultures were washed twice with 2 mL of 1x PBS warmed at 37°C and fixed with 1 mL of 10% buffered formalin. Fixed cultures were washed twice with PBS at room temperature and resuspended in 250 μL of PBS. Fixed cells were stored at 4°C for a maximum of one month until analysis.

*DBD-FISH* - DBD-FISH was performed on *M. tuberculosis*-infected and uninfected macrophages according to Vázquez-Gundín et al. (2000). Whole DNA probes were prepared from DNA isolated from human peripheral blood mononuclear cells and labelled using biotin-14-2′-deoxyuridine 5′-triphosphate (dUTP) using a commercial nick-translation reagent kit (Roche Diagnostics Corp., Indianapolis, IN, USA) according to Fernández and Gosálvez (2002). DNA probes were denatured at 73°C for 10 min and subjected to overnight hybridisation at 37°C. Slides were washed with 50% formamide/2′ SSC (pH 7) for 15 min and then with 2x SSC (pH 7) for 8 and 2 min in Tween 4 solution. A nonspecific antibody-blocking solution was applied for 5 min at 37°C prior to the detection of the hybridised signal. The hybridised DNA probe was detected after 30-min incubation with fluorescein isothiocyanate (FITC)-labelled avidin (1:400) (Roche Diagnostics Corp.). The slides were counterstained with 4′,6-diamidino-2-phenylindole (DAPI) (1 μg/mL) in Vectashield^®^ mounting medium (Vector Laboratories, Inc., Burlingame, CA, USA).

The integrated density (ID; segmented area of interest x grey level values obtained after background subtraction) was calculated using image analysis software (NIH Image Version 1.4.3.67) (Rasband 1997-2006). Fifty nuclei were examined for each sample.

*Fluorescence microscopy and digital image analysis* - All slides were analysed on a digital image analysis platform based on a Zeiss AxioPhot fluorescence microscope (Carl Zeiss, Göttingen, Germany) equipped with three low-pass band filters (green, red, and blue). The images were recorded using an AxioCam 16-bit black-and-white charge-coupled device in a 12-bit TIFF format. ID values obtained after background subtraction were calculated using Image J 1.4.3.6.7 analysis software (http://rsb.info.nih.gov/ij/). Fifty cell nuclei were examined for each sample.

*DNA fragmentation test* - *M. tuberculosis*-infected and uninfected macrophages were analysed by DNA fragmentation assay, based on the chromatin dispersion test described by Fernández and Gosálvez (2002) to determine DNA damage associated with mycobacteria infection. Slides were stained with DAPI (1 μg/mL) in Vectashield^®^ mounting medium (Vector Laboratories, Inc.) and analysed using fluorescence microscopy (Carl Zeiss). As a positive control, each experiment included uninfected macrophages treated with 1.3% hydrogen peroxide (H_2_O_2_) solution for 15 min to confirm the accessibility of the tested chemicals to the cell nucleus.

*Micronuclei assay* - The formation of MN in *M. tuberculosis*-infected macrophages was analysed using a standard protocol, as previously described (Fenech et al. 1999). MN were stained with propidium iodide (1 μg/mL) in Vectashield^®^ and 100 cells were scored for each experiment; two observers quantified the number of MN. In addition, nucleus abnormalities were analysed by epifluorescence microscopy (Carl Zeiss, Gottingen, Germany).

*Statistical analysis* - Mann-Whitney test was used for the fluorescence (area, intensity, and ID) analysis of cells after DBD-FISH. Student's *t*-test was used for the analysis of MN and nucleus abnormalities. A value of p < 0.05 was considered significant. All analyses were performed using IBM SPSS for Windows (v. 20.0; IBM Corp., Armonk, NY, USA).

## RESULTS

**Fig. 1:**
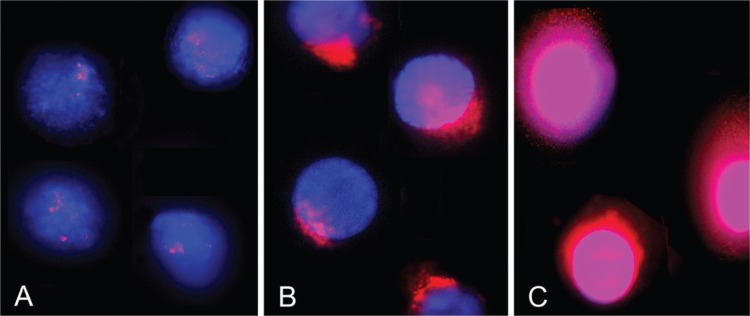
DNA breakage detection fluorescence in situ hybridisation (DBD-FISH) on infected human macrophages. Uninfected (A) and *Mycobacterium tuberculosis*-infected (B) human macrophages after DBD-FISH using whole-genome probe labelled with biotin stain; DAPI was used as a counterstain. Macrophages treated with hydrogen peroxide were used as the positive control (C).

*Mycobacterium tuberculosis (H37Rv) induces DNA breaks and ALS in human macrophages* - To determine the basal levels of DNA damage in uninfected macrophages, DBD-FISH technique was performed under mild alkaline denaturation conditions using a whole-genome DNA probe (Fig. 1A). *M. tuberculosis*-infected macrophages showed a significant increase in DNA breaks localised in blebs (Fig. 1B, Table).

**TABLE:** Fluorescence analysis in human macrophages infected with *Mycobacterium tuberculosis* after DNA breakage detection fluorescence in situ hybridisation (DBD-FISH) using a whole-genome DNA probe, biotin stain, and DAPI as a counterstain

	Fluorescence analysis (DBD-FISH)		
Group	Area (X ± SD)	Intensity(X ± SD)	ID (10^4^) (X ± SD)
Non-infected	601 ± 427+	150 ± 83+	9.01 ± 8.7+
Infected	2206 ± 942* +	176 ± 184* +	38.82 ± 46.7* +
Positive control	4221 ± 2739*	157 ± 134*	66.27 ± 58.2*

*: a significant difference (p < 0.001) was observed with respect to uninfected macrophages.

+: a significant difference (p < 0.05) was observed with respect to positive control.

The positive control for DNA damage treated with H_2_O_2_ showed higher ID values (Table). A strong fluorescence signal was uniformly distributed throughout the nucleus (Fig. 1C). These observation confirmed the accessibility of the tested chemicals to the cell nucleus.

**Fig. 2:**
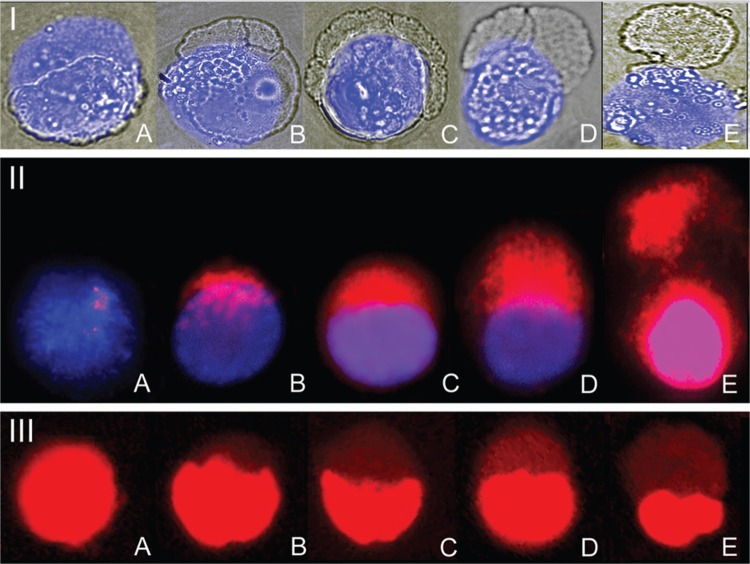
nuclear blebs in mycobacteria-infected macrophages. Panel I. Phase-contrast microphotographs of macrophages infected with *Mycobacterium tuberculosis* were overlapped with DAPI fluorescent staining images revealing peripheral vesicles in the nucleus (B-E). Uninfected macrophages (A). Panel II. DNA breakage detection fluorescence in situ hybridisation (DBD-FISH) on infected macrophages using whole-genome probe labelled with biotin and DAPI contrast showed an increase in FISH signal corresponding to bleb location (BD). Uninfected macrophages showed discrete fluorescence signals, considered as “constitutive or basal” (A). Dead cells (apoptosis/necrosis) showing strong fluorescence signal (E) were excluded. Panel III. Similar results were observed by DNA fragmentation test (A-E).

Phase-contrast microscopy analysis of infected macrophages showed peripheral nuclear blebs (Fig. 2, Panel I: B-D); these structures were absent in uninfected macrophages (Panel I: A). The decondensed form of chromatin and disruption of the nuclear membrane revealed a fuzzy veil-like structure (Panel I: E). For the infected macrophages, DNA damage started with the polarisation of the nuclear material (Panel I: E) and the fluorescence signal corresponded to bleb development (Panel II: B-D). The disruption of the nuclear membrane revealed a wide area but a weakly fluorescent signal (Panel II: E). Uninfected macrophages showed a discrete fluorescent signal in dispersed form (Panel II: A). DNA damage, determined by DNA fragmentation assay, confirmed the increase in DNA strand breaks in peripheral blebs (Panel III: B-E) as compared with uninfected macrophages (Panel III: A).

**Fig. 3:**
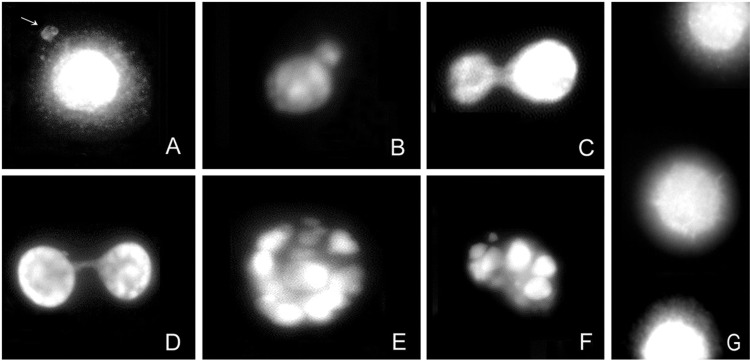
nuclear changes in *Mycobacterium tuberculosis*-infected macrophages. Micronuclei (MN) formation (A) and nuclear abnormalities such as nuclear bud (B), blebbing (C), nucleoplasmic bridge (D), nuclear shrinkage (E), and fragmentation formation (F) in *M. tuberculosis*-infected macrophages at 12 h were determined by propidium iodide stain. Uninfected macrophages were used as the control (G).

*M. tuberculosis infection induced MN formation and nuclear anomalies in macrophages* - We confirmed our results using other DNA damage markers. MN formation was determined in uninfected and *M. tuberculosis-*infected macrophages. For each condition, 100 cells were analysed for the detection of MN frequency. The extent of DNA damage was significantly higher (p < 0.05) in cells infected with *M. tuberculosis* (H37Rv) (13.0 ± 1.4 MN/100 cells) (Fig. 3A) as compared with uninfected cells (5.5 ± 2.1 MN/100 cells) In addition, other nuclear anomalies such as nuclear bud, blebbing, nucleoplasmic bridge, nuclear shrinkage, and fragmentation were observed in *M. tuberculosis*-infected cells (14.5 ± 2.1 nuclear anomalies/100 cells) (Fig. 3B-F) as compared with uninfected cells (4.0 ± 1.4 nuclear anomalies/100 cells). (Fig. 3G)

## DISCUSSION

As a chronic intracellular bacterial infection, TB is expected to induce DNA damage in the host through the induction of immune molecules and active metabolites of host immune cells and production of microorganism exo- and endotoxins, which act as chemical mutagens and generate chromosomal damage in affected cells (Rao et al. 1990, Rac et al. 1991).

Previous studies have demonstrated DNA damage in untreated TB patients using chromosomal abnormalities, MN techniques (Rao et al. 1990, Masjedi et al. 2000, da Silva et al. 2015), and comet assay (Selek et al. 2012).

However, Ekmekçi and Sayli (1995) reported that the frequency of chromosomal abnormalities and sister chromatid exchange showed no statistically significant differences between control patients and those before treatment with combination therapy.

In this study, we demonstrated that *M. tuberculosis* infection induced genomic instability in vitro, as evident from the appearance of several DNA damage markers (DNA break, MN, and nuclear abnormalities). Our results are in agreement with those reported by Mohanty et al. (2016), wherein a mouse model was used to reveal the increase in DNA damage by MN, nuclear abnormalities, comet assay, and H2AX.

DNA breaks and ALS in *M. tuberculosis*-infected macrophages were enriched in polarised blebs, as observed with DBD-FISH. It is interesting that the increase in the fluorescence intensity was associated with the size of the bleb (Fig. 2). These regions may be coupled to present phagocyte signals on the nuclear surface.

Macrophages infected with *M. tuberculosis* displayed MN formation. It is well established that MN may originate during anaphase from lagging acentric chromosome or chromatid fragments caused by misrepair of DNA breaks or unrepaired DNA breaks. Malsegregation of whole chromosomes at anaphase may lead to MN formation, owing to the hypomethylation of repeat sequences in centromeric and pericentromeric DNA, defects in kinetochore proteins or assembly, dysfunctional spindle, and defective anaphase checkpoint genes (Mohanty et al. 2016). The physiological role of MN formation is associated with mutagenic, genotoxic, or carcinogenic events. Recent studies have demonstrated the role of MN formation in DNA damage-induced immune activation (Harding et al. 2017).

We observed nuclear abnormalities associated with MN formation (nucleoplasmic bridge, nuclear buds, and blebbing) in *M. tuberculosis*-infected macrophages (da Silva et al. 2015, Mohanty et al. 2016). These abnormalities are indicative of the genome damage events and considered as biomarkers of genotoxic events and chromosomal instability (Fenech et al. 2011).

Macrophages infected with *M. tuberculosis* showed anomalies on chromatin condensation associated with the apoptosis process such as nuclear shrinkage and fragmentation (Banfalvi 2014). However, it is necessary to use apoptosis-specific markers to clarify whether *M. tuberculosis* infection contributes to apoptotic, biochemical, or morphological changes.

The activated macrophages express enzymes such as phagocyte oxidase (NOX2/gp91phox) and inducible nitric oxide synthase (iNOS), which generate reactive oxygen intermediates (ROI) and reactive nitrogen intermediates (RNI), respectively. The increase in the production of these molecules by macrophages is considered pathological, owing to their interferences with signalling pathways related to apoptosis and abilities to induce cell cycle arrest and cause severe damage to a wide range of molecules, including proteins, lipids, carbohydrates, and nucleic acids.

The main consequence of oxidative stress associated with TB infection is DNA damage, which may result in genomic instability. These damaged cells may be susceptible to elimination by apoptosis when the level of damage is very high (Chen et al. 2006, Lee & Kornfeld 2010). Loss of genomic stability is one of the most important aspects of mutagenesis and genetic changes associated with programmed cell death (Voskuil et al. 2011).

In pulmonary TB patients, Selek et al. (2012) reported an increase in DNA damage using the comet assay; this increase correlated with the total oxidant and anti-oxidant status and oxidative stress index (r = 0.69, p < 0.05; r = 0.48, p < 0.05, r = −0.47, p < 0.05; respectively).

Although the association of chronic inflammation and cancer is well documented, the causal relationship between TB infection and lung cancer is questionable. However, constant oxidative stress in lung cells serves as a source of free radicals, which may damage the DNA of pulmonary and circulating cells and contribute to the pathogenesis of lung cancer (Nalbandian et al. 2009).

The role of RNI in DNA damage in TB is controversial. Using a series of experiments in a mouse model, Mohanty et al. (2016) showed that *M. tuberculosis*-infected macrophages produce genomic instability associated with high levels of NO and ROI. In contrast, de Oliveira et al. (2012) studied lymphocytes of patients with pulmonary TB and failed to link DNA damage with iNOS gene expression, which is fundamental for nitric oxide production. However, DNA damage was suggested to be associated with other possible free radicals and oxidants (superoxide anion, nitrogen dioxide, H_2_O_2_, and hypochlorous acid).

Aside from the previous biomarkers used for the evaluation of DNA damage, DBD-FISH technique is a biomarker that deserves special attention, owing to its high sensitivity (Fernández et al. 1998). The present study analysed the overall genome using a whole-genome DNA probe. However, many different specific probes may be hybridised with the possibility of analysing chromosomal instability in macrophages within specific DNA sequence regions (Fernández et al. 1998, 2001a, b, Vázquez-Gundín et al. 2000).

In conclusion, our experiments support genomic instability in macrophages infected with *M. tuberculosis*. Further studies are needed to determine the association between ALS and oxidative as well as nitrosative stress during infection and evaluate the effects related to *M. tuberculosis* strain virulence and mycobacterial pathogenesis.
